# Predicting the bioactivity of 2-alkoxycarbonylallyl esters as potential antiproliferative agents against pancreatic cancer (MiaPaCa-2) cell lines: GFA-based QSAR and ELM-based models with molecular docking

**DOI:** 10.1186/s43141-021-00133-2

**Published:** 2021-03-10

**Authors:** Oluwatoba Emmanuel Oyeneyin, Babatunde Samuel Obadawo, Adesoji Alani Olanrewaju, Taoreed Olakunle Owolabi, Fahidat Adedamola Gbadamosi, Nureni Ipinloju, Helen Omonipo Modamori

**Affiliations:** 1 Theoretical and Computational Chemistry Unit, Adekunle Ajasin University, Akungba-Akoko, Ondo State Nigeria; 2 Department of Chemical Sciences, Adekunle Ajasin University, Akungba-Akoko, Ondo State Nigeria; 3 Department of Physics and Electronics, Adekunle Ajasin University, Akungba-Akoko, Ondo State Nigeria; 4 Chemistry and Industrial Chemistry Programmes, Bowen University, Iwo, Osun State Nigeria; 5 Department of Chemistry, University of Ibadan, Ibadan, Oyo State Nigeria; 6 Federal University of Agriculture, Makurdi, Benue State Nigeria

**Keywords:** 2-alkoxycarbonyl esters, Computer-aided drug design, Genetic function approximation, Extreme learning machine, Epidermal growth factor receptor, Molecular docking

## Abstract

**Background:**

The number of cancer-related deaths is on the increase, combating this deadly disease has proved difficult owing to resistance and some serious side effects associated with drugs used to combat it. Therefore, scientists continue to probe into the mechanism of action of cancer cells and designing novel drugs that could combat this disease more safely and effectively. Here, we developed a genetic function approximation model to predict the bioactivity of some 2-alkoxyecarbonyl esters and probed into the mode of interaction of these molecules with an epidermal growth factor receptor (3POZ) using the three-dimensional quantitative structure activity relationship (QSAR), extreme learning machine (ELM), and molecular docking techniques.

**Results:**

The developed QSAR model with predicted (*R*^2^_pred_) of 0.756 showed that the model was fit to be validated parameter for a built model and also proved that the developed model could be used in practical situation, *R*^2^ for training set (0.9929) and test set (0.8397) confirmed that the model could successfully predict the activity of new compounds due to its correlation with the experimental activity, the models generated with ELM models showed improved prediction of the activity of the molecules. The lead compounds (22 and 23) had binding energies of −6.327 and −7.232 kcalmol^−1^ for 22 and 23 respectively and displayed better inhibition at the binding sites of 3POZ when compared with that of the standard drug, chlorambucil (−6.0 kcalmol^−1^). This could be attributed to the presence of double bonds and the α-ester groups.

**Conclusion:**

The QSAR and ELM models had good prognostic ability and could be used to predict the bioactivity of novel anti-proliferative drugs.

## Background

In the world, pancreatic cancer (PC) is ranked the fourth highest cause of cancer-related deaths and the fourteenth most common cancer [1]. By the year 2030, it is predicted that it would become the second-most common cause of cancer-related deaths [2]. Pancreatic cancer is mainly divided into two types: (i) pancreatic adenocarcinoma (PA), the most common (85% of cases), arises in exocrine glands of the pancreas, and (ii) pancreatic neuroendocrine tumor (Pan-NET) which occurs in the endocrine tissue of the pancreas and less common [3]. MIAPaCa-2 and PANC-1 are two commonly used cancer cell lines for studies of PA [4]. PA is usually advanced at the time of diagnosis as it is relatively symptom-free [5]. With its poor prognosis, only 24% of people survive 1 year and 9% live for 5 years [3].

Late disease diagnosis, attainment of resistant characteristics, the paucity of effective therapies, and metastatic nature among others are some of the proposed reasons for the poor survival rate of PC patients [6]. Cytotoxic treatments presently used have failed, example of such is gemcitabine [7], with patients hardly survive more than 6 months after therapy, according to study [6]. 5-Fluorouracil, however, has been shown to be effective and boosts survival rate of patients [6]. In the last decades, the repurposing of approved drugs to treat cancer birthed drugs like celecoxib, metformin, sulindac, or TriFluoroPerazine (TFP). Although, there are limitations associated with them, an instance with TFP is that patients develop neurological side effects when it is used to treat cancer [8].

Some compounds such as methyl protodioscin could inhibit proliferation and promote apoptosis of MIAPaCa-2 cells [9]. A small-molecule, trisubstituted naphthalene diimide compound, is potent against PC cell line MIAPaCa-2 [10]. Another compound is ursolic acid, a popular anti-inflammatory and immunosuppressive agent which inhibited growth and induced apoptosis in a dose-dependent manner [11]. Due to this, the necessity of developing better and more effective therapeutics with improved activity at low concentrations that will ensure a longer survival rate and inhibit resistance against the MIAPa-Ca cancer cell lines cannot be overemphasized.

Quantitative structure activity relationship (QSAR) is one of the commonest approaches in ligand-based drug design process among Scaffold hopping, pseudo-receptor and pharmacophore modeling. The key goal of QSAR studies is to determine a mathematical model between the property under investigation, and one or more molecular descriptors [12–14]. Using the model, similar bioactivities of compounds not involved in the training set can be predicted from their structural descriptors [15]. Here, we used genetic function approximation (GFA) and ELM models to predict the bioactivity of the molecules under investigation.

ELM belongs to a class of feed forward neural networks characterized with a single hidden layer. The algorithm attains its uniqueness through random determination of the biases as well as the weights connecting hidden and input layers [16]. These features result into fast convergence, high training speed while the simple structure of ELM algorithm is maintained and thereby leads to enhanced computational efficiency coupled with impressive robustness [17].

Molecular docking analyzes the pose of molecules into the binding site of a macromolecular target and also probe into the mechanisms of binding of bioactive compounds and biological targets/receptors [3, 18, 19]. Therefore, this work is aimed at using QSAR to create a mathematical model from the selected training set of 2-alkoxycarbonylallyl esters derivatives (available in the literature) [20] to predict the activity of these compounds as anticancer agents against MIAPaCa-2 cancer cell lines and elucidate the interaction of these compounds as MIAPaCa-2 cancer cell line inhibitor via molecular docking studies.

## Methods

### QSAR Studies

#### Data collection

A series of twenty-four compounds of 2-alkoxycarbonylallyl esters [20] as a potential anticancer were collected from the literature. The curative activities of the compounds against given in IC_50_ (μM) were converted into their corresponding pIC_50_ values (i.e., - log IC_50_ = pIC_50_) in order to make the activities fit to a range of values and also to suit normal distribution curve. The experimental activities, pIC_50_ values, and compounds are presented (Table 1).

**Table 1:**
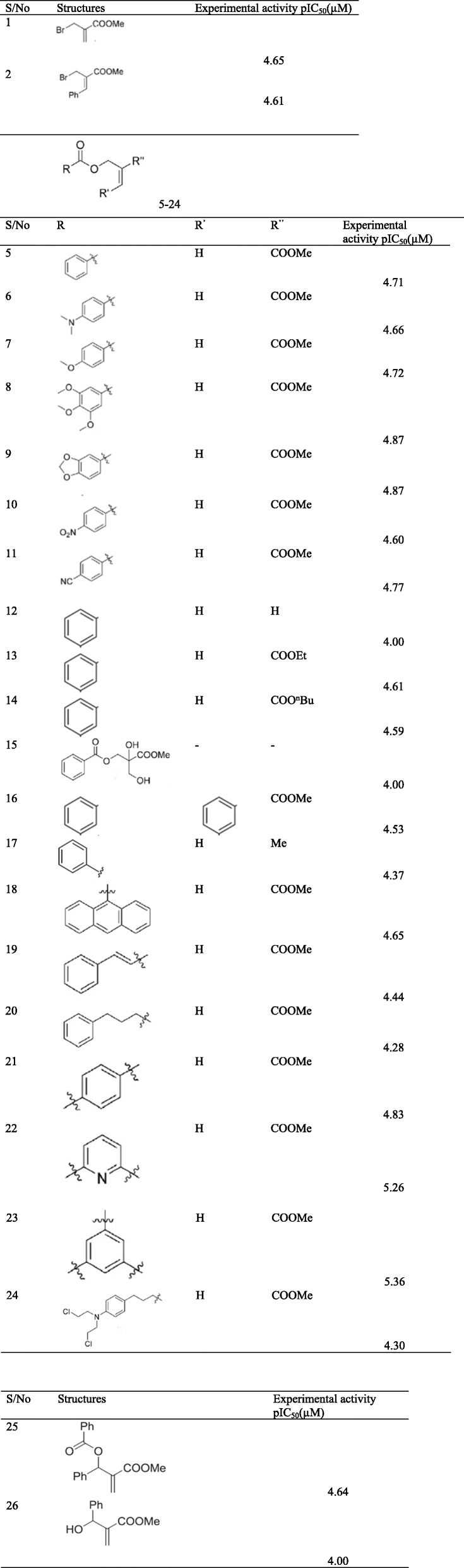
2-alkoxycarbonylallyl esters and their (pIC_50_)

The 2D structures of the compounds (Table 1) were drawn using ChemDraw Ultra 12.0 [21] and then saved as cdx file. The cdx file format of the compounds drawn was moved to the Spartan 14 software [22] and optimized using molecular mechanics with the molecular mechanics force field (MMFF) to generate their most stable conformers followed by a restricted hybrid Hartree Fock—density functional theory self-consistent field (HF-DFT SCF) calculation using Pulay’s DIIS and geometric direct minimization with the 6-31G* basis set [23]. The molecular structures were saved as sdf file format after optimizing the structures.

#### Molecular descriptor calculation

The optimized compounds were subjected into PaDEL-Descriptor software version 2.20 [24] in order to calculate the 1D, 2D, and 3D descriptors of the compounds. After removing salt, detecting tautomer and retaining the file name as molecule name, a total of 1875 descriptors were generated saved as Microsoft Excel Comma Separated value (csv) file.

### Mathematical description of the proposed ELM algorithm

ELM formalisms allow three layers with connected neurons. Pre-supposing that the number of neurons in the output, hidden, and input layers are indicated by *ξ*,*η* and *μ*, respectively with the threshold number of neurons for the input layer set as **I** = [*I*_1_, *I*_2_, .…*I*_*μ*_]^*T*^. Equation 1 defines the weights linking the hidden with input layer as represented by *ψ* while Eq. 2 presents the weights linking the hidden with output layer is *χ*.

1 $$ \boldsymbol{\uppsi} ={\left[\begin{array}{cccc}{\psi}_{11}& {\psi}_{12}& \dots & {\psi}_{1\eta}\\ {}{\psi}_{21}& {\psi}_{21}& \dots & {\psi}_{2\eta}\\ {}:& :& :& :\\ {}{\psi}_{\mu 1}& {\psi}_{\mu 2}& \dots & {\psi}_{\mu \eta}\end{array}\right]}_{\mu x\eta} $$

2 $$ \boldsymbol{\upchi} ={\left[\begin{array}{cccc}{\chi}_{11}& {\chi}_{12}& \dots & {\chi}_{1\xi}\\ {}{\chi}_{21}& {\chi}_{21}& \dots & {\chi}_{2\xi}\\ {}:& :& :& :\\ {}{\chi}_{\mu 1}& {\chi}_{\mu 2}& \dots & {\chi}_{\mu \xi}\end{array}\right]}_{\mu x\xi} $$

The experimental activities of the investigated compounds as well as the implemented descriptors are presented in matrix form as depicted in Eq. 3 and Eq. 4, respectively.

3 $$ \mathbf{D}={\left[\begin{array}{cccc}{d}_{11}& {d}_{12}& \dots & {d}_{1j}\\ {}{d}_{21}& {d}_{21}& \dots & {d}_{2j}\\ {}:& :& :& :\\ {}{d}_{\mu 1}& {d}_{\mu 2}& \dots & {d}_{\eta j}\end{array}\right]}_{\eta xj} $$

4 $$ A={\left[\begin{array}{cccc}{A}_{11}& {A}_{12}& \dots & {A}_{1j}\\ {}{A}_{21}& {A}_{21}& \dots & Aj\\ {}:& :& :& :\\ {}{A}_{\mu 1}& {A}_{\mu 2}& \dots & {A}_{\mu j}\end{array}\right]}_{\mu xj} $$

where *j* is the number of training samples.

With inclusion of a differentiable activation function *γ*(.)and network output *α*, the mathematical expression representing the working principles of ELM algorithm is presented in Eq. 5 [25, 26].

5 $$ \mathbf{P}\boldsymbol{\upchi } ={\boldsymbol{\upalpha}}^T $$

Equations 6 and 7 respectively presents the matrix form of hidden layer output (**P**) and network output.

6 $$ \mathbf{P}={\left[\begin{array}{cccc}\gamma \left({\psi}_1{d}_1+{I}_1\right)& \gamma \left({\psi}_2{d}_1+{I}_2\right)& \dots & \gamma \left({\psi}_{\mu }{d}_1+{I}_{\mu}\right)\\ {}\gamma \left({\psi}_1{d}_2+{I}_1\right)& \gamma \left({\psi}_2{d}_2+{I}_2\right)& \dots & \gamma \left({\psi}_{\mu }{d}_2+{I}_{\mu}\right)\\ {}:& :& :& :\\ {}\gamma \left({\psi}_1{d}_j+{I}_1\right)& \gamma \left({\psi}_2{d}_j+{I}_2\right)& \dots & \gamma \left({\psi}_{\mu }{d}_j+{I}_{\mu}\right)\end{array}\right]}_{jx\mu} $$

7 $$ \alpha ={\left[{\alpha}_1,{\alpha}_2,.\dots, {\alpha}_j\right]}_{\mu xj} $$

$$ {\boldsymbol{\upalpha}}_k=\left[\begin{array}{c}{\alpha}_{1k}\\ {}{\alpha}_{2k}\\ {}:\\ {}{\alpha}_{\mu k}\end{array}\right]={\left[\begin{array}{c}\sum \limits_{i=1}^{\mu }{\lambda}_{i1}\varphi \left({\boldsymbol{\upsigma}}_k{\mathbf{r}}_i+{z}_i\right)\\ {}\sum \limits_{i=1}^{\mu }{\lambda}_{i2}\varphi \left({\boldsymbol{\upsigma}}_k{\mathbf{r}}_i+{z}_i\right)\\ {}:\\ {}\sum \limits_{i=1}^{\mu }{\lambda}_{i\xi}\varphi \left({\boldsymbol{\upsigma}}_k{\mathbf{r}}_i+{z}_i\right)\end{array}\right]}_{\xi}\left(k=1,2,\dots, j\right) $$

Minimization of ‖**Pχ − α**^**T**^‖ yields Eq. 8.

8 $$ \boldsymbol{\upchi} ={\mathbf{P}}^{+}{\boldsymbol{\upalpha}}^{\mathbf{T}} $$

while **P**^+^stands for the Moore-penrose generalized inverse of **P**.

## Computational details

### QSAR methods

#### Normalization and data pre-treatment

The descriptors were normalized (Eq. 9) [27] and pre-treated using the Data Pre-treatment software [28].

9 $$ X=\frac{X_1-{X}_{\mathrm{min}}}{X_{\mathrm{max}}-{X}_{\mathrm{min}}} $$

where *X*_1_ is the descriptor’s value for each molecule, *X*_min_ and *X*_max_ are minimum and maximum value for each descriptor. This is done in order to filter descriptors with redundant data, highly correlated data, reduce colinearity thereby improving the performance of the model.

#### Data division

The pre-treated dataset was split into training and test sets by employing Kennard and Stone’s algorithm [29]. The training set, 70% of the data sets (16 compounds) was used to build the model and validated internally while 30% of the data sets (8 compounds) were used to externally validate the built model.

#### Model development

Material studio 2017 software was used to build the model employing the GFA method while fixing the biological activities (pIC_50_) as the dependent variable and the physiochemical properties (descriptors) as the independent variables.

#### Internal validation of model

The training compounds were validated internally using material studio. The validation parameters include the following:

##### Friedman’s lack of fit (LOF)

The models generated were appraised using LOF to obtain their fitness score (Eq. 10) [30]

10 $$ LOF=\frac{SEE}{{\left(1-\frac{c+\left(d\times p\right)}{M}\right)}^2} $$

SEE, the standard error of estimation; *p*, the number of descriptors used; *d* is a user-defined smoothing parameter, *c* is the number of model terms, and *M* is the number of compound in the training set [31].

For a good model, SEE value must be low, it is given as (Eq. 11):

11 $$ SEE=\sqrt{\frac{{\left({Y}_{\mathrm{exp}}-{Y}_{\mathrm{pred}}\right)}^2}{n-p-1}} $$

##### where n is the number compounds that made up the training set, Y_exp_ is experimental activity and Y_pred_ is the predicted activity in the training set                  The correlation coefficient (*R*^2^)

This is the mostly used for internal assessment of QSAR models. The *R*^2^ (Eq. 12) is expected to be very close to 1 to generate a good model.

12 $$ {R}^2=1-\frac{\sum {\left({Y}_{\mathrm{exp}}-{Y}_{\mathrm{pred}}\right)}^2}{\sum {\left({Y}_{\mathrm{exp}}-{Y}_{\mathrm{training}}\right)}^2} $$

where *Y*_training_ is the mean of the experimental activity.

##### Adjusted (*R*^2^)

 (Eq. 13) value varies directly with the increase in number of descriptors, it is important because it measures the reliability and stability of a model unlike *R*^2^.

13 $$ {R}_{\mathrm{adj}}^2=\frac{\left({R}^2-p\right)\times \left(n-1\right)}{n-p-1} $$

##### The cross validation coefficient $\left({Q}_{cv}^2\right)$

The strength of the QSAR model was cross-validated (Eq. 14).

14 $$ \left({Q}_{cv}^2\right)=1-\left\{\frac{\sum {\left({Y}_{\mathrm{pred}}-{Y}_{\mathrm{exp}}\right)}^2}{\sum {\left({Y}_{\mathrm{exp}}-{Y}_{\mathrm{training}}\right)}^2}\right\} $$

Where *Y*_training_, *Y*_exp_, *and Y*_pred_ were defined earlier

##### External validation of the model

The built model was validated externally by the *R*^2^_predicted_ value. The *R*^2^_predicted_ value is the most commonly used parameter to validate a built model. The *R*^2^_test_ is defined by (Eq. 15):

15 $$ {R}^2=1-\frac{\sum {\left({Y_{\mathrm{pred}}}_{\mathrm{test}}-{Y_{\mathrm{exp}}}_{\mathrm{test}}\right)}^2}{\sum {\left({Y_{\mathrm{pred}}}_{\mathrm{test}}-{\overline{Y}}_{\mathrm{training}}\right)}^2} $$

#### Statistical analysis of the descriptor

##### Variance inflation factor

VIF (Eq. 16) measures the multicolinearity of the descriptors among each other and also measures the degree at which one descriptor correlates with the others in a model.

16 $$ \mathrm{VIF}=\frac{1}{1-{R}^2} $$

*R*^2^ is the multiple correlation coefficient between all variables used in the model. If the VIF = 1, it signifies that there is no intercorrelation in each variable, and if it is between 1 and 5, it is acceptable. VIF value > 10 makes the model unstable.

##### Mean effect

This correlates the effect of a particular molecular descriptor on the activities of the compounds. A change in the descriptors’ values improves the activity of the compounds. It is defined (Eq. 17):

17 $$ \mathrm{Mean}\ \mathrm{Effect}=\frac{\ {B}_j{\sum}_i^n{D}_j}{\sum_j^m\left(\ {B}_j{\sum}_i^n{D}_j\right)} $$

where *B*_*j*_ and *D*_*j*_ are the *j*-descriptor coefficient in the model and the values of each descriptor in training set, while *m* and *n* stand for the number of molecular descriptors and number of molecules in a training set respectively. Therefore, the mean effect of each descriptor used in building the model was calculated to assess the significance of the model [32].

##### Evaluation of the applicability domain of the model

This is a vital step in establishing that the model is good to make predictions [33]. The leverage approach [34] was applied here. Leverage of a given chemical compound *hi*, is defined as (Eq. 18):

18 $$ hi={X}_i{\left({X}^TX\right)}^{-1}{X}_i^T $$

*X*_*i*_ is the training compounds matrix of *i*. *X* is the m × k descriptor matrix of the training set compound and *X*^*T*^ is the transpose matrix of *X* used to build the model. The warning leverage, h* (Eq. 19) is the limit of normal values for *X* outliers:

19 $$ {h}^{\ast }=3\frac{\left(k+1\right)}{n} $$

where *n* and *k* are the descriptors and the training set compounds respectively.

##### Quality assurance of the model

The validation parameters test the strength, dependability, and predictiveness of a built-in QSAR model. Consequently, Table 2 establishes general minimum specifications for both internal and external validation parameters to validate a QSAR model [34]. The list of descriptors, their descriptions, and dimension are presented (Table 3).

**Table 2:** Generally recommended values for the validation parameters for a built model

Parameter	Definition	Recommended value
*R*^2^	Coefficient of determination	≥0.6
*P*_(95%)_	Confidence interval at 95% confidence level	<0.05
${Q}_{cv}^2$	Cross validation coefficient	≥0.5
*R*^2^ -${Q}_{cv}^2$	Difference between R^2^ and ${Q}_{cv}^2$	<0.3
*N*_(ext & test set)_	Minimum number of external test set	≥5
$c{R}_p^2$	Coefficient of determination for Y-randomization	≥0.5

**Table 3:** List of descriptors, their constructors, description and dimension used in building the QSAR model

S/No	Name	Description	Dimension
1	ATSC3c	Centered Broto-Moreau autocorrelation—lag 3/weighted by charges	2D
2	MATS5p	Moran autocorrelation—lag 5/weighted by polarizabilities	2D
3	minHBint5	Minimum E-state descriptors of strength for potential hydrogen bonds of path length 5	2D
4	ETA_Shape_P	Shape index P	2D

### ELM-based models

The modeling and simulation that leads to the development of the proposed ELM-based models was implemented on MATLAB. The descriptors to the model as well as the experimental activities of the investigated compounds were initially randomized prior to separation into training and testing sets of data in the ratio of 4:1, respectively. Randomization enhances even distribution of data points and improves computational efficiency of the algorithm. The training dataset was employed in generating weights which are further validated using testing set of data. The procedures for the computational implementation of the proposed ELM-based models are detained as follow:

- **Step I:** *Initial random number generation:* Pseudo random number that controls the randomly generated biases as well as the weights linking the hidden with input layers were initiated with the aid of seeding in MATLAB.
- **Step II:** *Optimum activation function and corresponding hidden nodes:* One activation function was selected after the other within a pool of functions which include sine function, hardlim function, and sigmoid function triangular basis function among others. Hidden nodes were optimized within search space of 1 to 100.
- **Step III:** *Computation of hidden layer output matrix:* Equation (6) was implemented on training dataset for the calculation of the hidden layer output matrix.
- **Step IV:** *Output weight computation:* with the aid of the least square solution to the set of the generated linear system of equations, the output weights were computed.
- **Step V:** *Evaluation of the developed models:* The developed models during the training phase of the simulation were evaluated using testing set of data. The performance of the model was assessed using four different performance measuring parameters which include MAE, RMSE, CC, and MAPD. The models characterized with lowest error (MAE, RMSE, and MAPD) and highest CC was saved as the best models. The hyper-parameters of the best model as well as the weights were also saved for ensuring reproducibility of the results.

### Molecular docking and ADME/Tox screening

#### Protein and ligands preparation

**Fig. 1:**
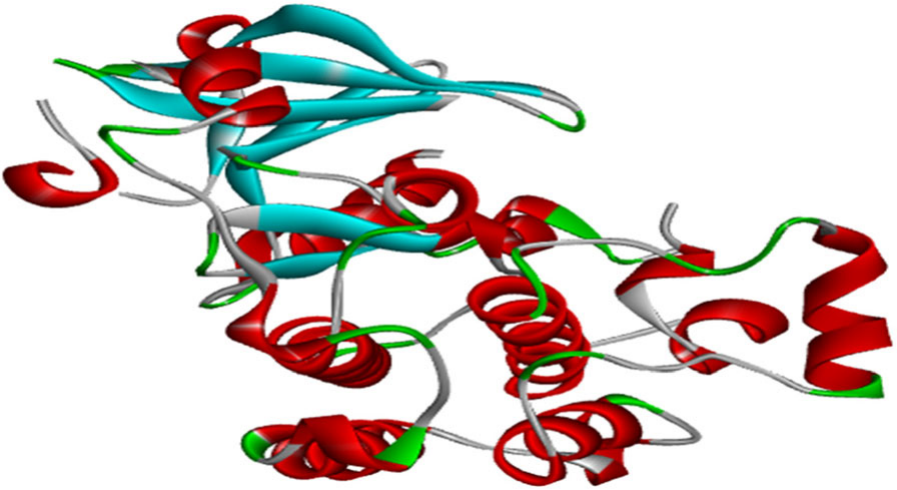
Structure of the receptor, 3POZ

The 3D crystal structure of an epidermal growth factor receptor (EGFR), a kinase domain (PDB ID: 3POZ) was downloaded from the protein data bank (Fig. 1) [35]. The receptor has two ligands (O3P) and sulfate ion (SO_4_) in its active sites [36]. The receptor was chosen because it is an EGFR which best works with cancer lines caused by cell proliferation and its high resolution of 1.5 Å [36, 37]. The downloaded protein was refined using the Protein Preparation Wizard [37, 38] by assigning charges and bond orders with the removal of water molecules and addition of hydrogens to the heavy atoms. Energy minimization was done using OPLS3 [38, 39].

#### Ligand preparation

The lead molecules (22 and 23) were selected for docking and prepared using ligand preparation (ligprep) in Schrödinger Suite 2017-1 with an OPLS3 force field [39] in order to create three dimensional geometries and to assign proper bond orders. Epik 2.2 in Schrödinger Suite at pH 7.0 ± 2.0 was used to generate the ionization state [40]. They, alongside with a standard drug, chlorambucil were docked at the active site of the receptor (*x* = 20.3785826772, *y* = 32.8299212598, and *z* = 14.8903149606).

#### ADME/Tox screening

The compounds were further screened for their drug-likeness by calculating the absorption, distribution, metabolism, excretion, and toxicity (ADME/Tox) properties using the QikProp program [41]. These properties check drug fitness and save cost involved in bioassay studies [42]. Descriptors like molecular weight (Mw), number of hydrogen bond donors (donorHB) and number of hydrogen bond acceptors (acceptHB), solvent accessible surface area (SASA), octanol/water partition coefficient (QPlog Po/w), and value for serum protein binding (QPlogKHsa) were considered (Table 11).

## Results

### QSAR results of 2-alkoxycarbonylallyl esters

A QSAR model was developed to predict the activity of MIAPaCa-2 cancer cell lines in 2-alcoxycarbonylallyl esters (pIC50). The multiple linear regression (MLR) for the built model is shown in Eq. 20.

20 $$ \mathbf{pIC}\mathbf{5}\mathbf{0}=\left(2.115051910\times \boldsymbol{ATSC}\mathbf{3}\boldsymbol{c}\right)-\left(0.917421961\times \boldsymbol{MATS}\mathbf{5}\boldsymbol{p}\right)-\left(\ 0.160590092\times \boldsymbol{minHBint}\mathbf{5}\right)+\left(724494169\times \boldsymbol{ETA}\_\boldsymbol{Shape}\_\boldsymbol{P}\right)+3.419964012 $$

The list of descriptors, their constructors, description, and dimension used in building the QSAR model are reported in Table 3. Table 4 represents the comparison between experimental activity (pIC50), predicted activity (pIC50), and residual of the developed model which is used in validating the model externally as reported in Tables 5 and 6.

**Table 4:** Comparison of experimental activity (pIC_50_), predicted activity (pIC_50_), and residual of developed model

S/No	Experimental activity (pIC_50_)	Predicted activity (pIC_50_)	Residual
1	4.9115	4.9067	0.0048
2	4.0000	4.0070	−0.0070
5	4.4743	4.4012	0.0731
6^a^	4.2248	4.3582	−0.1334
7^a^	4.5452	4.4373	0.1078
8	4.6548	4.6486	0.0062
9^a^	4.3706	4.4557	−0.0852
10^a^	4.6185	4.5560	0.0625
11	4.5424	4.5056	0.0368
12^a^	4.0000	3.8942	0.1058
13	4.1748	4.1651	0.0097
14	4.0000	3.9974	0.0026
15	4.0000	4.0032	−0.0032
16	4.0000	3.9531	0.0469
17	4.1392	4.2393	−0.1002
18^a^	4.1395	3.9735	0.1660
19	4.1914	4.1859	0.0055
20^a^	4.0000	3.7604	0.2396
21	4.8573	4.8269	0.0304
22	5.4750	5.5102	−0.0353
23	5.3344	5.3283	0.0061
24	4.0786	4.0943	−0.0156
25^a^	4.0232	4.0193	0.0039
26	4.1232	4.1841	−0.0609

^a^Test set

**Table 5:** External validation of developed model

Name	ATSC3c	MATS5p	minHBint5	ETA_Shape_P	*Y*_exptest_	*Y*_predtest_
10	0.1896	−0.2790	0.0000	0.2778	4.6185	4.5560
12	0.0761	−0.0651	0.0000	0.1471	4.0000	3.8942
18	0.0244	−0.2706	0.0000	0.1471	4.1395	3.9735
20	0.0417	−0.2081	1.6445	0.1887	4.0000	3.7604
25	0.0817	−0.1618	0.0000	0.1613	4.0232	4.0193
6	0.1136	−0.2585	0.0000	0.2672	4.2248	4.3582
7	0.1352	−0.3068	0.0000	0.2609	4.5452	4.4373
9	0.1967	−0.3069	0.0000	0.1961	4.3706	4.4557

**Table 6:** Calculation of the predicted *R*^2^ of developed model

*Y*_predtest_ − *Y*_exptest_	*Y*_predtest_ − *Y*_exptest_	${\overline{Y}}_{\mathrm{training}}$	${Y_{\mathrm{pred}}}_{t\mathrm{est}}-{\overline{Y}}_{\mathrm{training}}$	${Y_{\mathrm{pred}}}_{\mathrm{test}}-{{\overline{Y}}_{\mathrm{training}}}^2$
−0.0625	0.0039	4.3699	0.2486	0.0618
−0.1058	0.0112	4.3699	−0.3699	0.1368
−0.1660	0.0276	4.3699	−0.2304	0.0531
−0.2396	0.0574	4.3699	−0.3699	0.1368
−0.0039	0.0000	4.3699	−0.3467	0.1202
0.1334	0.0178	4.3699	−0.1451	0.0210
−0.1078	0.0116	4.3699	0.1753	0.0307
0.0852	0.0073	4.3699	0.0007	0.0000

∑(*Y*_predtest_ − *Y*_exptest_)^2^ = 0.1368

$$ \sum {\left({Y_{\mathrm{pred}}}_{\mathrm{test}}-{\overline{Y}}_{\mathrm{training}}\right)}^2=0.5605 $$

$$ {R}_{\mathrm{Pred}}^2=1-\frac{0.1368}{0.5605}=0.7560 $$

**Fig. 2:**
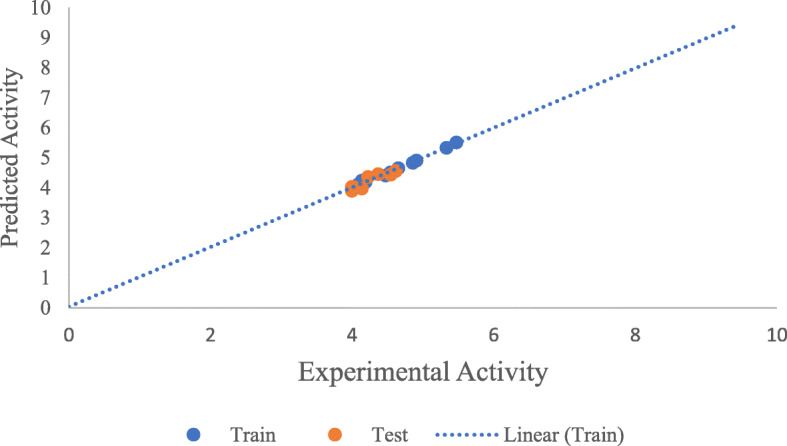
Predicted activity vs experimental activity for both training set and test set

**Fig. 3:**
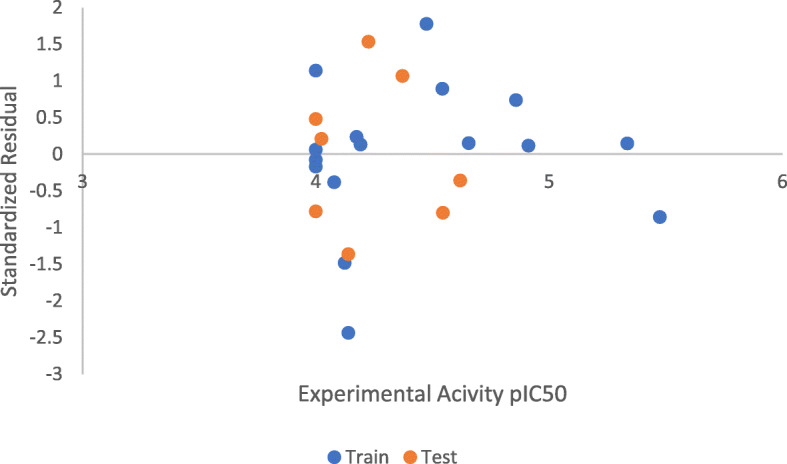
Standardized residual vs experimental activity pIC_50_

**Fig. 4:**
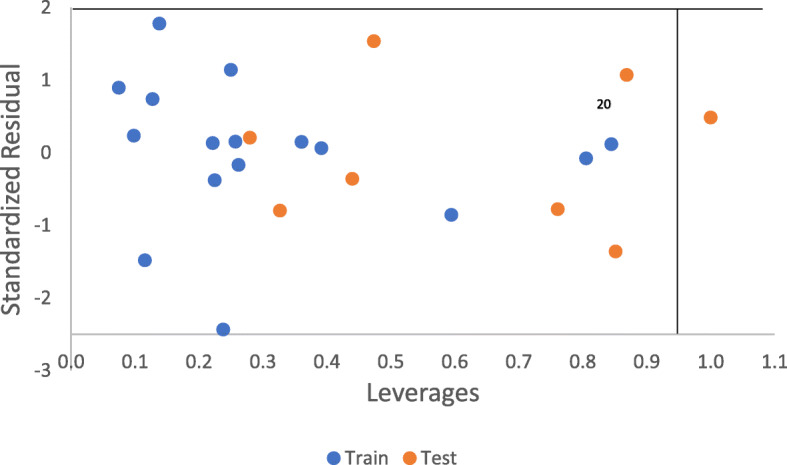
Williams plot of standardized residual vs against leverages

Table 7 presents the Pearson’s correlation, mean effect, and variance inflation factor of the descriptors used in building the model. The experimental activity is plotted against the predicted activity for both training set, and test set shown in Fig. 2. The scatter plot between standardized residual activity and experimental activity which explains the randomness of the activities on both negative and positive sides of *y*-axis is in Fig. 3. While Fig. 4 presents the Williams plot of standardized residual against leverages for the developed model.

**Table 7:** Pearson’s correlation matrix, VIF and ME of the descriptors used in the built model

Descriptors	ATSC3c	MATS5p	minHBint5	ETA_Shape_P	VIF	ME
ATSC3c	1	−0.5467	0.5262	0.1569	2.6933	0.3872
MATS5p	−0.5467	1	0.0824	−0.2116	1.9799	0.2565
minHBint5	0.5262	0.0824	1	−0.0068	1.8967	−0.0886
ETA_Shape_P	0.1569	−0.2116	−0.0068	1	1.0506	0.4449

### Comparison of the estimates of developed QSAR and ELM-based models

**Fig. 5:**
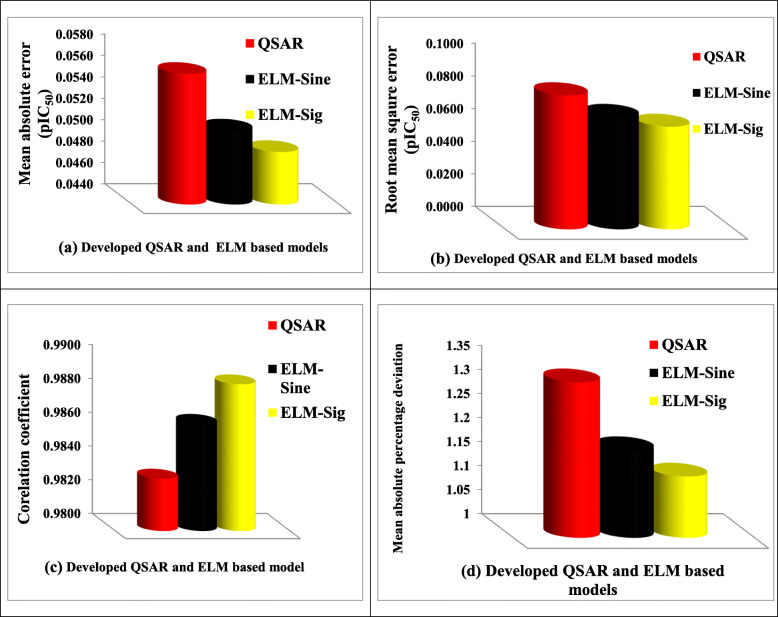
Comparison of the performance of the developed models using (**a**) MAE, (**b**) RMSE, (**c**) CC, and (**d**) MAPD performance measuring parameters

The performance of the developed ELM-based models was compared with that of QSAR model using four different performance measuring parameters including mean absolute error (MAE), root mean square error (RMSE), correlation coefficient (CC), and mean absolute percentage deviation (MAPD). The outcomes of the comparison are presented in Table 9 and Fig. 5a-d. The comparison of the outcomes of each of the developed models with inclusion of percentage error for each of the investigated compounds is presented in Table 10.

**Table 8:** Validation parameter for the built model

S/N	Validation parameter	Value
1	Friedman LOF	0.010894
2	*R*-squared	0.992869
3	Adjusted *R*-squared	0.990276
4	Cross validated *R*-squared	0.987188
5	Significant Regression	Yes
6	Significance-of-regression *F* value	382.902997
7	Critical SOR *F* value (95%)	3.403505
8	Replicate points	0
9	Computed experimental error	0
10	Lack-of-fit points	11
11	Min expt. error for non-significant LOF (95%)	0.035818
12	*R*^2^_pred_	0.756016

**Table 9:** Performance comparison of the developed QSAR and ELM-based models

	MAE (pIC_50_)	RMSE (pIC_50_)	CC	MAPD
QSAR	0.0562	0.0822	0.9831	1.323677
ELM-sine	0.0509	0.0689	0.9863	1.182579
ELM-sig	0.0489	0.0629	0.9887	1.127731

**Table 10:** Comparison of the estimates of the developed models

S/N	Experimental activity (pIC_50_)	QSAR predicted activity (pIC_50_) (this work)	%error	ELM-Sine predicted activity (pIC_50_) (this work)	% Error	ELM-Sig predicted activity (pIC_50_) (this work)	% Error
1	4.9115	4.9067	0.0977	4.9385	0.5493	4.8448	1.3589
2	4.0000	4.0070	0.1750	3.9999	0.0019	3.9996	0.0106
5	4.4743	4.4012	1.6338	4.4120	1.3919	4.3969	1.7295
6	4.2248	4.3582	3.1575	4.3714	3.4692	4.3472	2.8982
7	4.5452	4.4373	2.3739	4.4601	1.8727	4.4277	2.5852
8	4.6548	4.6486	0.1332	4.6534	0.0291	4.6555	0.0148
9	4.3706	4.4557	1.9471	4.5178	3.3690	4.4533	1.8923
10	4.6185	4.5560	1.3533	4.5696	1.0597	4.5461	1.5679
11	4.5424	4.5056	0.8101	4.5379	0.0996	4.4983	0.9703
12	4.0000	3.8942	2.6450	3.9358	1.6052	3.9817	0.4565
13	4.1748	4.1651	0.2323	4.2014	0.6378	4.1751	0.0065
14	4.0000	3.9974	0.0650	4.0626	1.5646	4.0472	1.1800
15	4.0000	4.0032	0.0800	4.0157	0.3930	4.0405	1.0118
16	4.0000	3.9531	1.1725	3.9822	0.4460	3.9595	1.0130
17	4.1392	4.2393	2.4183	4.2094	1.6951	4.2496	2.6660
18	4.1395	3.9735	4.0101	3.9754	3.9637	4.0114	3.0952
19	4.1914	4.1859	0.1312	4.1513	0.9557	4.1897	0.0408
20	4.0000	3.7604	5.9900	3.9952	0.1190	4.0102	0.2543
21	4.8573	4.8269	0.6259	4.8418	0.3188	4.8159	0.8514
22	5.4750	5.5102	0.6429	5.4205	0.9955	5.4652	0.1781
23	5.3344	5.3283	0.1144	5.3572	0.4279	5.3681	0.6321
24	4.0786	4.0943	0.3849	4.0783	0.0063	4.1023	0.5817
25	4.0232	4.0193	0.0969	4.1032	1.9879	4.0618	0.9585
26	4.1232	4.1841	1.4770	4.1819	1.4232	4.1691	1.1120
**MAPD**			**1.3237**		**1.1826**		**1.1277**

### Molecular docking

**Fig. 6:**
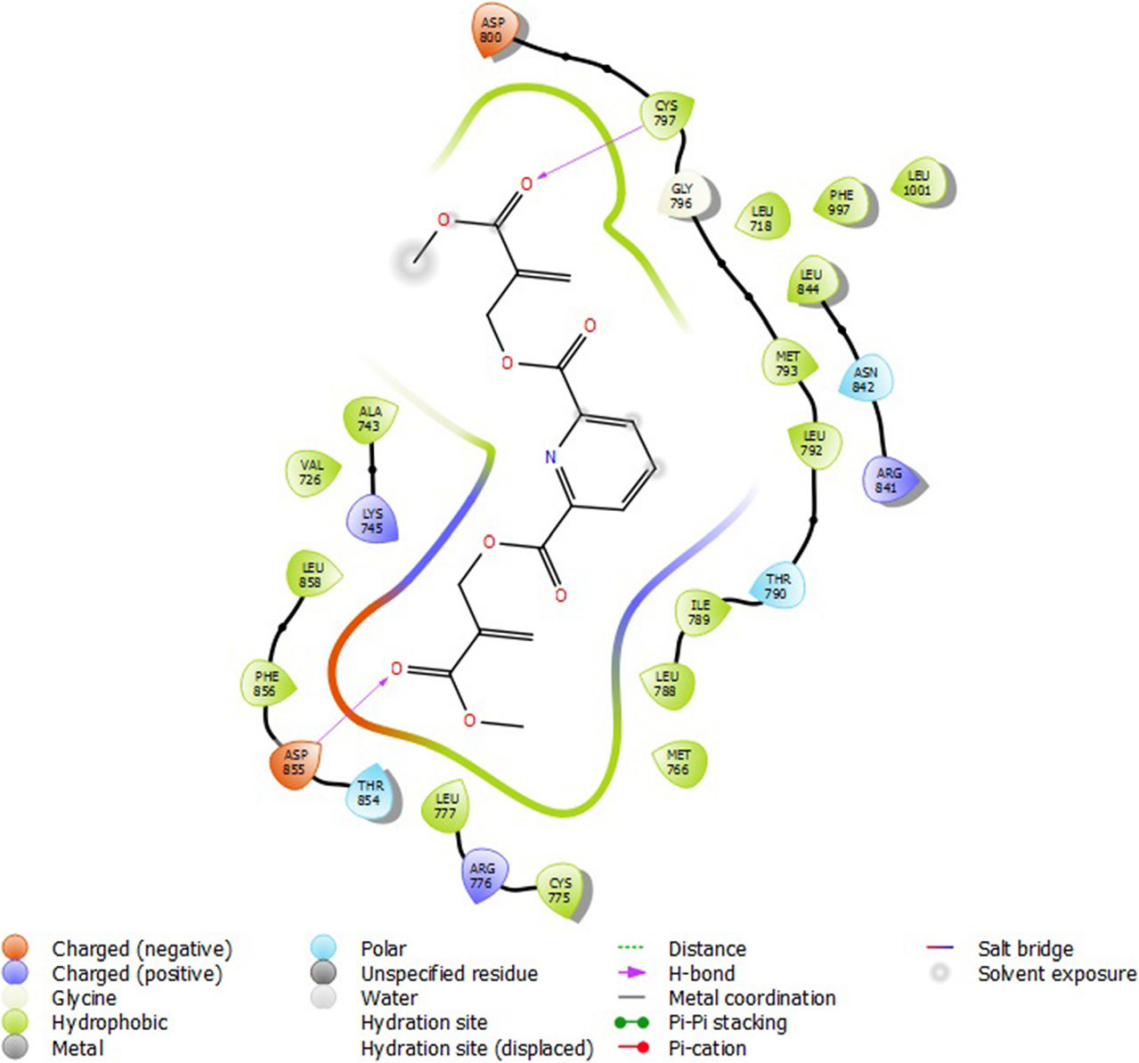
Compound 22 with 3POZ

**Fig. 7:**
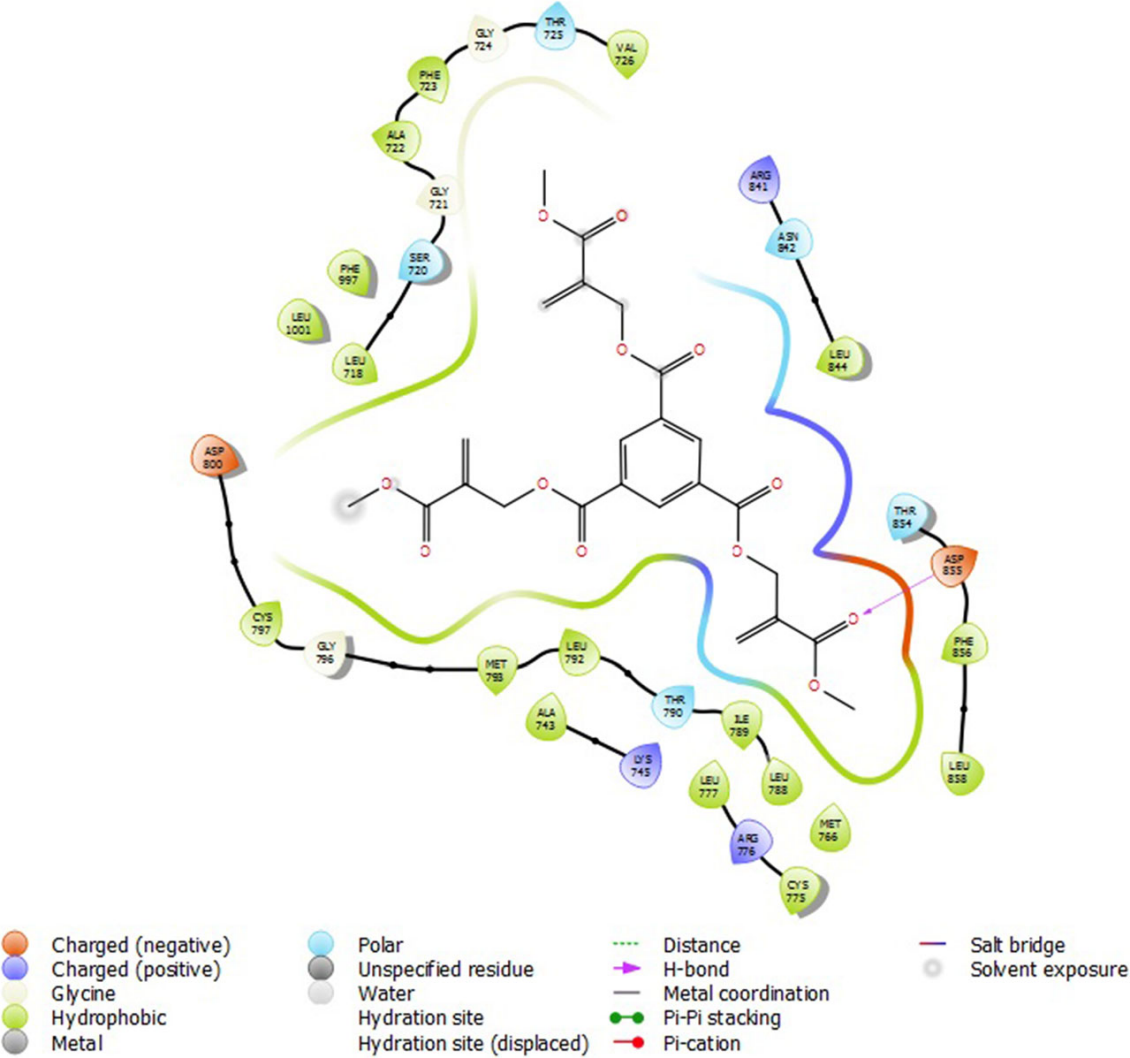
Compound 23 with 3POZ

Molecular docking studies were carried out to understand the mechanism of action of some 2-alkoxycarbonylallyl esters against pancreatic cancer (MiaPaCa-2) cell line targeting the epidermal growth factor receptor (EGFR). The structure of the prepared receptor is shown in Fig. 1. The reference drug (chlorambucil) and lead compounds (22 and 23) were docked with the epidermal receptor growth factor (3POZ) to elucidate the interaction and the binding mode. The binding affinities and ADME/Tox properties of chlorambucil and the lead compounds (22 and 23) are presented in Table 11. While the interactions between the epidermal receptor growth factor (3POZ) with the chlorambucil and lead compounds (22 and 23) are presented respectively in Figs. 5, 6, and 7.

**Table 11:** Binding affinities and ADME/Tox properties of some of the lead molecules and chlorambucil

Molecules	Binding energy (kcalmol^**−1**^)	Mw	SASA	donorHB	accptHB	QPlogPo/w
22	−6.327	363	604	0	9	1.34
23	−7.232	504	877	0	12	1.92
Chlorambucil	−5.826	304	581	1	3	4.60

## Discussion

### QSAR results of 2-alkoxycarbonylallyl esters

Four different models were generated using the genetic function approximation technique. The first model was selected to be the best model due to its statistical significance and the fact that it satisfied the recommended standard for a stable and reliable model as outlined (Table 2).

2D descriptors played a vital role in predicting the activity of new molecules that can inhibit against MIAPaCa-2 cancer cell line. The positive coefficient of **ATSC3c, MATS5p**, **minHBint5**, and **ETA_Shape_P** descriptors in the model inferred that increase in the coefficient of the descriptor will improve the activity (pIC_50_) of **2-alkoxycarbonylallyl esters** against MIAPaCa-2 cancer cell line. Hence, to design potent compounds with high pIC_50_ value, the positive coefficient of the descriptors will have to be increased.

The low values of residual recorded in Table 4 affirmed that there is high correlation between the experimental activities and predicted activities. The value of *R*^2^_pred_ (0.7560) signifies that the model has passed the minimum recommended value for validating parameter for a built model presented in Table 2. The idea that once the *R*^2^_predicted_ value is considered satisfied, the remaining parameters will also be satisfied is not always true as additional statistical analyses can help validate the built model such as variance inflation factor (VIF) and mean effect (ME). The closer the value of *R*^2^_test_ to 1.0, the better the stability the model generated. Therefore, in prediction of the behavior of a new compound, the stability will take into account model reliability.

The Pearson’s correlation, ME, and VIF of the descriptors are presented in Table 7. The low correlation values ≤ 0.5 in most descriptors inferred that the descriptors do not correlate with one another. This ascertains that there is no bias in the prediction made by the model. The mean effect of the descriptors shown in Table 7 signifies the effect of molecular descriptors on the activity pIC_50_ of the compounds. Thus, the order of decreasing effect is **ETA_Shape_P > ATSC3c > MATS5p**> **minHBint5**.

The experimental activity was plotted against predicted activity for both training set and test set is reported in Fig. 2. The high value of correlation coefficient *R*^2^ for training set (0.9929) and test set (0.8397) confirmed that the model can successfully predict the activity of a new compound due to its correlation with the experimental activity. The randomness of the activities on both negative and positive sides of *y*-axis shown on the scatter plot between standardized residual activity and the experimental activity reported in Fig. 3 confirmed the built model was free from systematic error. To discover outliers and influential compounds in the built model, the standardized residual activity for the entire data set was plotted against the leverages. Williams plot (Fig. 4) confirmed that there was only one influential compound (20) with leverage value of 1.00 which is greater than the warning value (*h*= 0.9375).

### Comparison of the estimates of developed QSAR and ELM-based models

Using MAE metric, the ELM-Sig built model was more efficient than ELM-Sine and QSAR models with a performance improvement of 4.09% and 14.92% respectively. The model performed higher with a performance improvement of 9.53% and 30.68% of the RMSE performance assessment parameter respectively. For CC and MAPD metrics, the developed ELM-Sig model performed better than ELM-Sine and QSAR models with performance enhancement of 0.24% and 0.57% as well as 4.86% and 17.38%, respectively. The developed ELM-Sine model also performed better than QSAR-based model with improved performance of 10.41%, 19.30%, 0.33%, and 11.93%, respectively using MAE, RMSE, CC, and MAPD as performance measuring parameters.

From Table 10, the results of the developed ELM-sig model show persistence closeness with the measured values. The superiority of the developed ELM-based model over the QSAR model can be attributed to the intrinsic feature of ELM algorithm in approximating non-linear and complex relationship linking the descriptors to the target. Ability of sigmoid activation function to approximate well is reveled from the performance of ELM-Sig model over that of ELM-Sine model.

### Molecular docking

The lead compounds (22 and 23) showed good docking score in the receptor’s active sites and are better than chlorambucil, a standard drug (Table 11). Compound 22 (−6.327 kcalmol^−1^) showed conventional hydrogen bond with CYT 737 and ASP 855 from its carbonyl oxygen from the α-ester group (Fig. 5). Compound 23 (−7.232 kcalmol^−1^) also showed conventional hydrogen bond with ASP 855 from its carbonyl oxygen from the α-ester group (Fig. 6). Chlorambucil (−5.826 kcalmol^−1^) showed no conventional hydrogen bond with any of the amino acid groups (Fig. 7). The lead molecules have better activity than chlorambucil as reported by Conor et al. [20]; the bonds responsible for their bioactivity according are the double bond and the α-ester groups. The ADME/Tox properties (Table 11) showed that these compounds are fit as drugs, according to Lipinski’s rule of five [19, 43, 44].

**Fig. 8:**
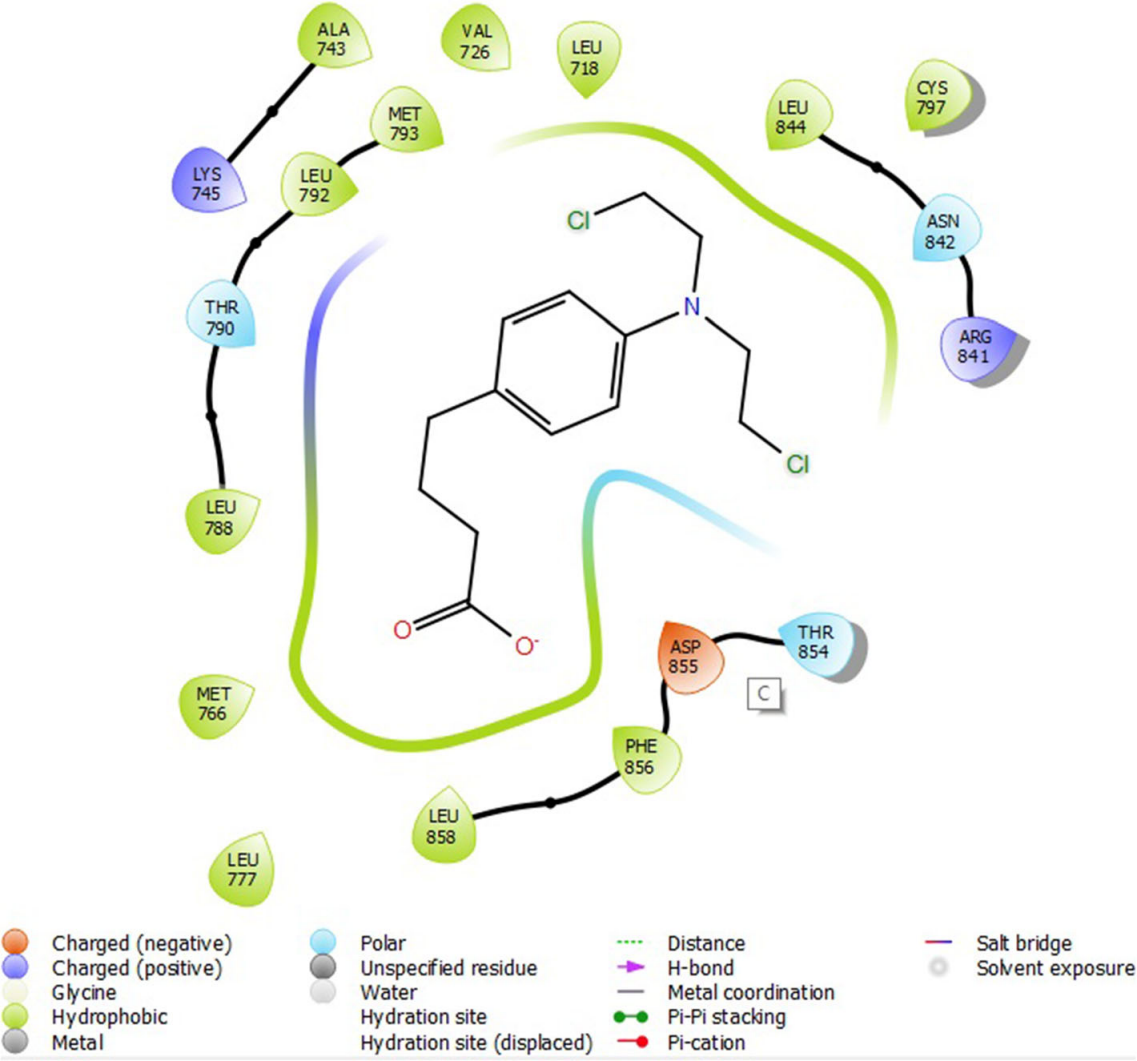
Chlorambucil with 3POZ

## Conclusion

In this study, a GFA model, together with two EML models were used to predict the potential activity of 2-alkoxycarbonylallyl esters as anticancer against MIAPaCa-2 pancreatic cancer cell lines while also probing into the mechanisms of interaction of the lead compounds against an epidermal growth factor receptor kinase domain, 3POZ via molecular docking approach. The GFA model generated was thoroughly validated; this model was compared to with ELM-Sig model and ELM-Sine model, with the ELM-Sig model proving the best in bioactivity prediction of these molecules. Binding of the lead compounds with the receptor showed they had better inhibitory potentials than chlorambucil. In the 2D interaction diagrams, it was seen that the compounds bind to the receptor predominantly through the hydrogen bond interaction and also mainly the carbonyl oxygen atoms of the α-esther group were responsible for their interaction, which is in line with what was observed in the experiment.

## Data Availability

Structures, experimental activity, molecular descriptors, and statistical analysis during the study are included in this published article.

## Abbreviations

GFA: Genetic function approximation
EGFR: Epidermal growth factor receptor
ELM: Extreme learning machine
PA: Pancreatic adenocarcinoma
Pan-NET: Pancreatic neuroendocrine tumor
TFP: TriFluoroPerazine
QSAR: Quantitative structure activity relationship
